# Using nuclear magnetic resonance to assist in calculating the structure of Fischer-Tropsch lubricant

**DOI:** 10.3389/frma.2025.1415831

**Published:** 2025-05-01

**Authors:** Hu Li, Tengfei Jiang, Yushan Jiang, Xuemei Liang, Xingyu Su, Liangcheng An, Nana Fan, Likun Yang, Linhua Song

**Affiliations:** ^1^Ningxia Coal Industry Co., Ltd., China Energy Group, Yinchuan, China; ^2^College of Chemistry and Chemical Engineering, China University of Petroleum (East China), Qingdao, China

**Keywords:** Fischer-Tropsch lubricant base oils, nuclear magnetic resonance technology, structural parameters, physico-chemical properties, conformational relationships

## Abstract

Research on Fischer-Tropsch (FT) synthetic lubricant base stock products is expected to fill a global gap in high-quality lubricants. However, the lack of identifiable characteristic functional groups in their pure hydrocarbon compositions makes it difficult to accurately analyze their compositions using existing methods. In this study, we propose a method combining nuclear magnetic resonance (NMR) and high-temperature gas-phase analysis to achieve a simple and accurate calculation of the structural information of lubricant base oils. Four structural parameters of FT lubricant base oils—namely, the average carbon number (C^*^), the number of branched chain nodes (B), the degree of branching (BI), and the structural index (BC^*^)—were successfully calculated using a series of empirical equations. Subsequently, we correlated the molecular structure parameters of the oils with their density, viscosity, viscosity index, and condensation point. Effective fitting equations were developed and quantitatively verified. Studies have shown that the physicochemical properties of lubricant base oils can be related to the structural parameters BC^*^ or BBC^*^. BBC^*^ fits better, with an *R*^2^ value of up to 0.91 or more, except for the condensation point. Density correlates well with viscosity, with a calculation error of <5%. This method of calculating the structural information of lubricant base oils can be applied to the structural determination of many hydrocarbon base oil molecules, while the simulation equations can simultaneously be used as a reference for the structure-function relationship of distillate base oils.

## 1 Introduction

Lubricants are widely used in modern industry and are essential for the smooth operation of machinery and equipment. The performance of lubricants is closely related to the base oils, which are divided into three main categories: mineral base oils, synthetic base oils, and vegetable oil base oils (Haus et al., 2003; Premuzic and Lin, 1999; Shah et al., 2021). Conventional lubricant base oils are usually derived from petroleum, which is a non-renewable fossil fuel. To a certain extent, it is difficult to achieve the development and utilization of renewable and environmentally friendly lubricants (Rajabi et al., 2020; Freschi et al., 2022). The simple product structure makes Fischer-Tropsch lubricant base stocks easy to formulate. They are also free of sulfur and nitrogen compounds and low in aromatic hydrocarbons. Their widespread use can effectively reduce dependence on petroleum-based lubricant base oils while meeting the environmental requirements for cleaner oils. This aspect of the product is unmatched by petroleum-based products (Neuner et al., 2021). Meanwhile, the product has a high viscosity index and low evaporation loss, which makes it suitable for the production of lubricant base oils with ultra-high viscosity indexes. It can meet the increasingly stringent demand for automotive antifreeze lubricants and offers competitive advantages in the production of high value-added products, such as high-grade lubricant base oils and high melting point paraffin waxes (Zhang et al., 2018).

Despite the apparent simplicity in composition and structure, FT lubricant base oils exhibit complexity in several aspects, including the diversity of alkane structures, the proportion of ortho-isomerized hydrocarbons, the number of branched chains, and their positions. These internal structures significantly influence key physicochemical properties such as density, viscosity, and viscosity index (Kobayashi et al., 2009, 2005; Noh et al., 2017). In turn, these properties are closely linked to the performance and service life of lubricants. Therefore, a comprehensive understanding of the specific composition and structure of FT lubricant base oils is essential to ensure their safe usage (Sharma and Gandhi, 2008; Narayanasarma et al., 2023).

Currently, many analytical methods can be used to determine the composition and structure of oils, such as gas chromatography (GC), gas chromatography-mass spectrometry (GC-MS), liquid chromatography-mass spectrometry (LC-MS), thermal analysis, and infrared spectroscopy (IR; Wang and Zhang, 2007; Shi et al., 2010; Scheuremann et al., 2011; Agarwal and Porwal, 2023; Chen et al., 2023). However, these techniques face challenges in effectively analyzing the composition of FT lubricant base oils. Specifically, the presence of numerous corrective molecules in base oils complicates accurate analysis of the oil composition and mass spectrometry (MS), as it requires high resolution and standardized reference materials. Furthermore, GC sampling can be challenging due to the high boiling point of FT-based lubricant base oils (Lee et al., 2022).

NMR technology is capable of overcoming the issues mentioned above and has a wide range of applications in calculating the structural information of oil products. Based on NMR analysis, various parameters can be obtained, including average carbon number, degree of branching, and methyl content of oil products (Sarpal et al., 1998; Erro et al., 2020; Jameel et al., 2016). For example, Sarpal et al. (1997) used ^13^C-NMR to determine the hydrocarbon composition of base oils produced by different processes, obtaining precise structural parameters. In addition to standard NMR, multi-pulse 1D and 2D NMR spectroscopy were employed to calculate the molecular structure of hydrocracked base oils, providing the average number of methyl branches per molecule within the range of 3.6–4.0 (Sarpal et al., 1996). It's worth noting that this study involved simulating characteristic chemical shifts of different structural oil components for formula derivation, presenting certain limitations in specific practical applications.

On this basis, this paper presents a comprehensive method for analyzing and calculating the structure of FT-based lubricant base oils using nuclear magnetic resonance and high-temperature gas chromatography simulated distillation. Reliable fitting equations for constitutive relationships are developed to accurately predict the density, viscosity, viscosity index, and condensation point of FT-based lubricant base oils by correlating a large amount of structural and physicochemical property data. This innovative approach simplifies the testing process and provides a practical theoretical framework for predicting the physicochemical properties of industrial lubricants.

## 2 Materials and methods

### 2.1 Raw materials

Different types of FT lubricant base oils were provided by Ningxia Coal Industry Co., Ltd, and the samples were numbered as L-1–L-10. L-1–L-5 are the products after hydroisomerization of Fischer-Tropsch oils. L-6–L-10 are hydroisomerized and subsequently refined products. The class analog oils were provided by Ningxia Coal Industry Co., Ltd, and the samples were numbered as N-1–N-27. The N-1–N-11 series are narrow-cut fraction samples prepared from L-3 via narrow boiling-range fractional distillation with 30°C intervals (e.g., N-1: 270–300°C, N-2: 300–330°C). The N-12–N-27 series are narrow-cut fraction samples prepared from L-5 via narrow boiling-range fractional distillation with 30°C intervals, following the same methodology as applied to N-1–N-11. Deuterated chloroform was purchased from Shanghai McLean Biochemical Science and Technology Co, Ltd, and the purity of the reagent was 99.8%.

### 2.2 Experimental instruments

The density of the samples was measured at 20°C using a density tester (BN-013) from Dalian Bangneng Petroleum Instrument Co. Viscosity and viscosity index were determined using an Anton Paar fully automated kinematic viscometer (SVM 1001). The base oil viscosity was measured at 40 and 100°C and the viscosity index was obtained by calculation. The condensation point was tested using an automatic petroleum product condensation point tester (JSR0919) from Hunan Jinshi Petrochemical Instrument Co. NMR tests include ^1^H-NMR, ^13^C-NMR and DEPT spectroscopy. Among them, the NMR DEPT spectra include both 90° and 135°. Measurements were carried out using a Bruker Ascend NMR spectrometer at 400 M. Carbon number distribution was tested using a high temperature simulated distillation apparatus (JAS-63198-0200-130) from Joint Analytical Systems GmbH, Germany.

### 2.3 Research route

**Figure 1:**
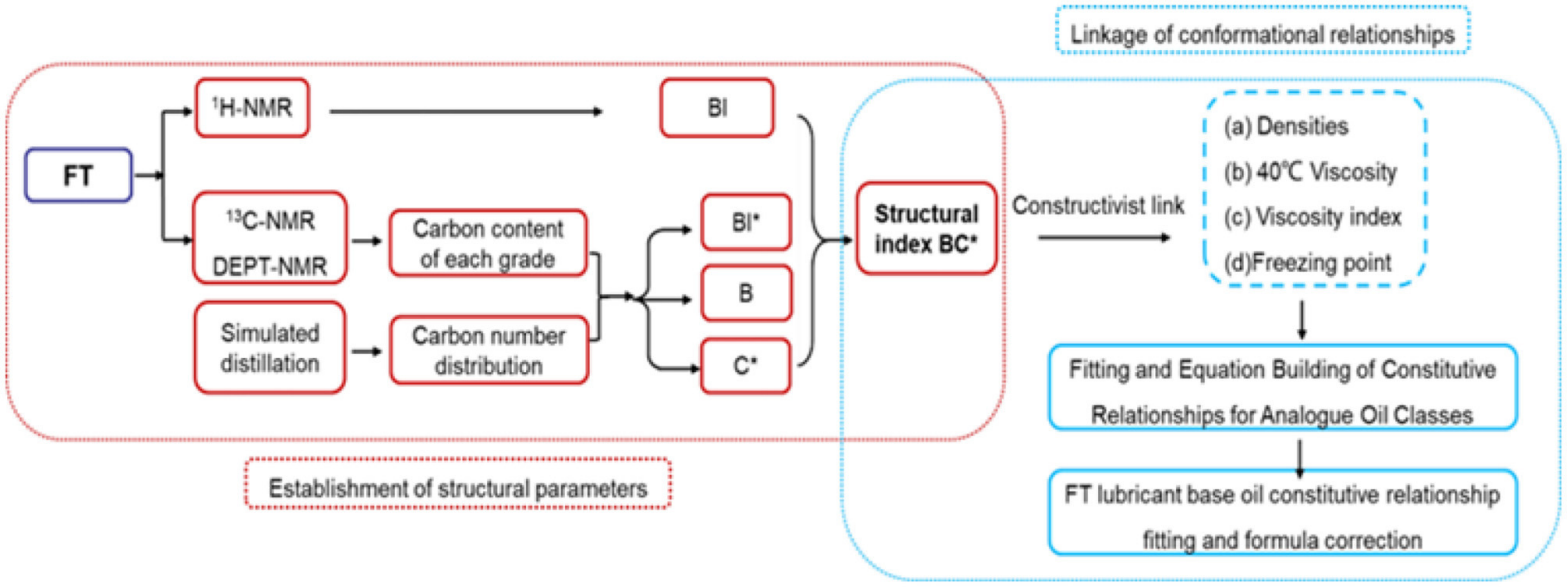
Research flow for the derivation of structural parameters.

The overall process is shown in Figure 1. The research idea of this study can be divided into two parts: the first part is the establishment of structural parameters and the second part is the establishment of constitutive relations. First, ^1^H-NMR, ^13^C-NMR, DEPT NMR-assisted high-temperature simulations and carbon number distributions were used to obtain important structural parameters such as the average carbon number (C^*^), the number of branched nodes (***B***), and the degree of branching (***BI***). The class analog oils ^a^ are used to establish the structure-activity relationship between structural parameters and properties. A series of fitting equations were obtained, which were combined to find the most representative structural parameters (BC^*^)**. **Then we use BC^*^ to establish the correlation between structure and density, viscosity, viscosity index and condensation point, derive a series of empirical formulas and validate them, and then extend them to establish the constitutive relationship of FT-based lubricant base oils. Thus, the physical and chemical properties of the structural components can be accurately predicted.

## 3 Results and discussion

### 3.1 Establishment of structural parameters

#### 3.1.1. Derivation of structural parameters

**Figure 2:**
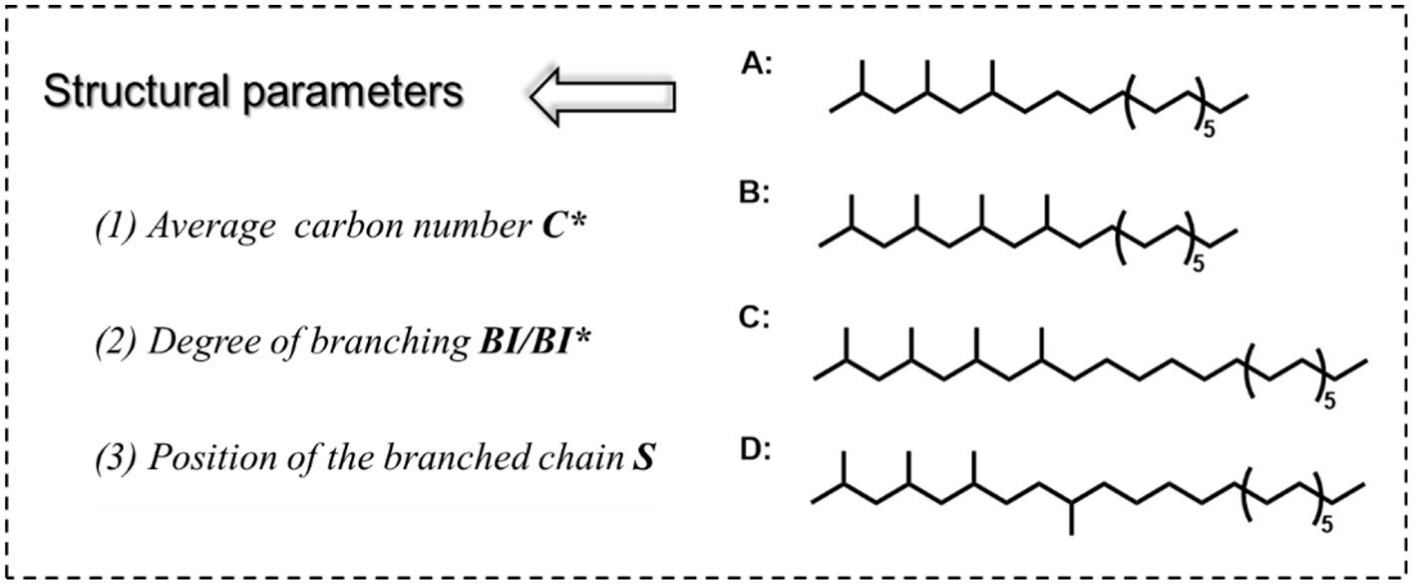
Schematic diagram of structural parameters of the oil model.

FT lubricant base oils consist of hydrocarbons with different lengths of carbon chains and are relatively homogeneous. It can neither be divided into chain hydrocarbon, cyclic hydrocarbon, and aromatic hydrocarbon like conventional petroleum base oil, nor has different structural units like ester base oil. Therefore, there are difficulties in how to characterize the structure of a large number of hydrocarbon mixtures with informative parameters. Firstly, to better characterize the structure of FT lubricant base oils, we propose three typical parameters were proposed: C^*^, BI, and the position of the branched chain (***S***). The three parameters together affect the composition and proper-ties of the base oil. Single-molecule A, B, C, and D are used as the model structure of the oil, as shown in Figure 2. A and B have the same C^*^ and different ***BI***; B and C have the same ***BI*** and different C^*^, C, and D have the same ***BI*** and C^*^, and ***S*** is different.

The average carbon number is expressed as the number of carbons in each component after averaging. The degree of branching is expressed in two ways: one quantifies the *BI* in terms of the proportion of hydrogen at each level, and the other calculates the BI^*^ in terms of the proportion of carbon at each level, both of which are approximately equal. As shown in Supplementary Figure S1, the branched chain position (*S*) can exist in a variety of forms in the structure of the FT lubricant base oil blend. The influence of each is masked by the others, so the structural composition of lubricant base oils can be described in detail in terms of the average carbon number and degree of branching. The structural parameters of the model compounds A, B, C, D, and the mixed ABC fractions in Figure 2 were calculated using the following equations:


(1) $$C^{*}=C_{\;A}^{*}\times n_{A}+C_{\;B}^{*}\times n_{B}+\ldots C_{\;X}^{*}\times n_{x}$$

(2) $$B=2\times \frac{C_{CH}}{C_{CH3}}$$

(3) $$BI\;=\;\frac{H_{CH3}/3}{H_{CH+CH2}/2}\times 100\%$$

(4) $$BI^{*}=\frac{C_{CH3}}{C_{CH+CH2}}\times 100\%$$

Where, ${\text{C}}_{\text{x}}^{*}$ indicates the average carbon number of the *x* component; *n*_*x*_ represents the proportion of the X component; ***B*** indicates the number of branched nodes, i.e., the number of branched chains; *BI* indicates the degree of branchedness calculated quantitatively from the ratio of primary to secondary and tertiary hydrogen sums; BI^*^ indicates the degree of branchedness quantitatively calculated as the ratio of primary to secondary and tertiary carbon sums.

From the calculation results summarized in Table 1, it can be observed that there is always an approximately equal relationship between ***BI*** and BI^*^ for both single-component and multi-component calculations, with the BI^*^ values being slightly smaller than the *BI* values. This discrepancy arises because it is not possible to distinguish between H_CH_ and H_CH2_ in the ^1^H-NMR spectra, and the formula takes the average value, which results in slightly smaller denominators. Therefore, the expression for BI^*^ is relatively more accurate.

**Table 1:** Calculation of component structure parameters.

**Designation**	***C*** ^*^	***BI*** **/%**	***BI*** ^*^/%	***S***
A	25	21.28	20.00	S7
B	25	35.29	31.58	S10
C	30	27.27	25.00	S10
D	30	27.27	25.00	S5/S7
Mixture^a^	27	30.05	27.36	–

^a^20% of component A + 40% of component B + 40% of component C.

#### 3.1.2 Structural parameters corresponding to the NMR calculation formula

FT lubricant base oils are composed of alkanes with different carbon chain lengths, and the key parameters for describing their structural information are the average carbon number C^*^ and the degree of branching ***BI***. NMR technology has been developed as an effective method for characterizing their structural composition. Both ^1^H-NMR and ^13^C-NMR are used to obtain accurate information on C^*^ and ***BI***, providing a basis for calculating the structural parameters of FT lubricant base oils. The degree of branching is calculated with reference to conventional oil branching methods (Zhang et al., 2019), and the ^1^H-NMR spectrum can be used to obtain the Equation 10. The formula for the average carbon number C^*^ is derived from the integral data of ^13^C-NMR, as shown in Supplementary Figure S1 and Supplementary Table S2. It is well known that the integral data of carbon spectra are not as accurate as those of hydrogen spectra, but interpreting many parameters in oil research requires the use of integral carbon spectra data (Sarpal et al., 1998; Erro et al., 2020). To correct the accuracy of the carbon spectra for use in FT lubricant base oils, the degree of branching calculated using ^1^H-NMR is labeled as *BI*, and the degree of branching calculated using ^13^C-NMR is labeled as BI^*^, with the results from both methods being approximately equal. Combined with the average chain lengths calculated in the high-temperature gas phase, as shown in Supplementary Figure S2, these findings indicate that the integral data of the ^13^C-NMR spectra are suitable for structural calculations of FT lubricant base oils. We calculated and corrected the degree of branching using both ^1^H-NMR and ^13^C-NMR formulas, resulting in the following reasonable calculation equation:

- Average carbon number C^*^:

(5) $$tMe\;=\;100\frac{\left[I_{a\sim b}\right]}{I_{T}}$$

(6) $$bMe\;=\;100\frac{\left[I_{c\sim d}\right]}{I_{T}}$$

(7) $$NP\;=\;100\frac{\left[3I_{e}+I_{f\sim g}\right]}{I_{T}}$$

(8) $$IP\;=\;NP\frac{\left[I_{a\sim b}-I_{e}\right]}{I_{e}}$$

(9) $$C^{*}\;=\;2\frac{NP+IP}{tMe}$$

Where, t-Me indicates the terminal methyl content, the content occupied by the methyl group located in the terminal position. Class analog oils a ~ b: 10–15.8 ppm; FT lubricant base oils a ~ b: 13.8–14.6 ppm. b-Me indicates the branched methyl content the content occupied by the intermediate-substituted methyl group. Class analog oils c ~ d: 15.8–21.0 ppm + 28.0 ppm; FT lubricant base oils c ~ d: 19.2–22.8 ppm. NP denotes ortho hydrocarbon carbon content calculated using ^13^C-NMR. Class analog oil e: 32.0 ppm, f ~ g: 29.3–30.3 ppm; FT lubricant base oils e: 32.0 ppm, f ~ g: 29.2–30.7+37.2 ppm. IP denotes isoparaffin carbon content calculated using ^13^C-NMR. Class analog oils a ~ b: 10–15.8 ppm, e: 32.0 ppm; FT lubricant base oils a ~ b: 13.8–14.6 ppm, e: 32.0 ppm. C^*^ denotes the average carbon number of the entire FT lubricant base oils. The symbol I in the equation denotes the integration interval on the ^13^C-NMR spectrum, and I_T_ represents the area-integrated counts at chemical shifts of 0–50 ppm.

(2) Degree of branch chaining ***BI*** (BI^*^):


(10) $$BI\;=\frac{I_{\left(A\sim C\right)}*2}{I_{\left(C\sim D\right)}*3}\;$$

(11) $$B\;=\;2\times \frac{bMe}{tMe}$$

(12) $$BI^{*}=\;\frac{B+2}{C^{*}-2-1.5\times B}$$

Where ***BI*** denotes the degree of branchedness calculated quantitatively using the ratio of hydrogen at each level in ^1^H-NMR; ***B*** denotes the number of branched nodes, the number of substituted branched chains; BI^*^ denotes the degree of branchedness quantitatively calculated using the ratios of carbon at each level in ^13^C-NMR. I denotes the area of integration on the ^1^H-NMR spectrum. Values range from A ~ C: 0.7–1.0 ppm for the integrated area of the peaks at the chemical shifts where the primary hydrogens are located; C ~ D: 1.0–1.7 ppm for the integrated area of the peaks at the chemical shifts where the secondary and tertiary hydrogens are located.

(3) Structural parameters BC^*^ and BBC^*^:


(13) $$BC^{*}\;=\;B\times C^{*}$$

(14) $$BBC^{*}\;=\;B\times B\times C^{*}$$

Where ***B*** denotes the number of branched nodes, the number of substituted branched chains, C^*^ denotes the average carbon number of the entire FT lubricant base oils.

**Figure 3:**
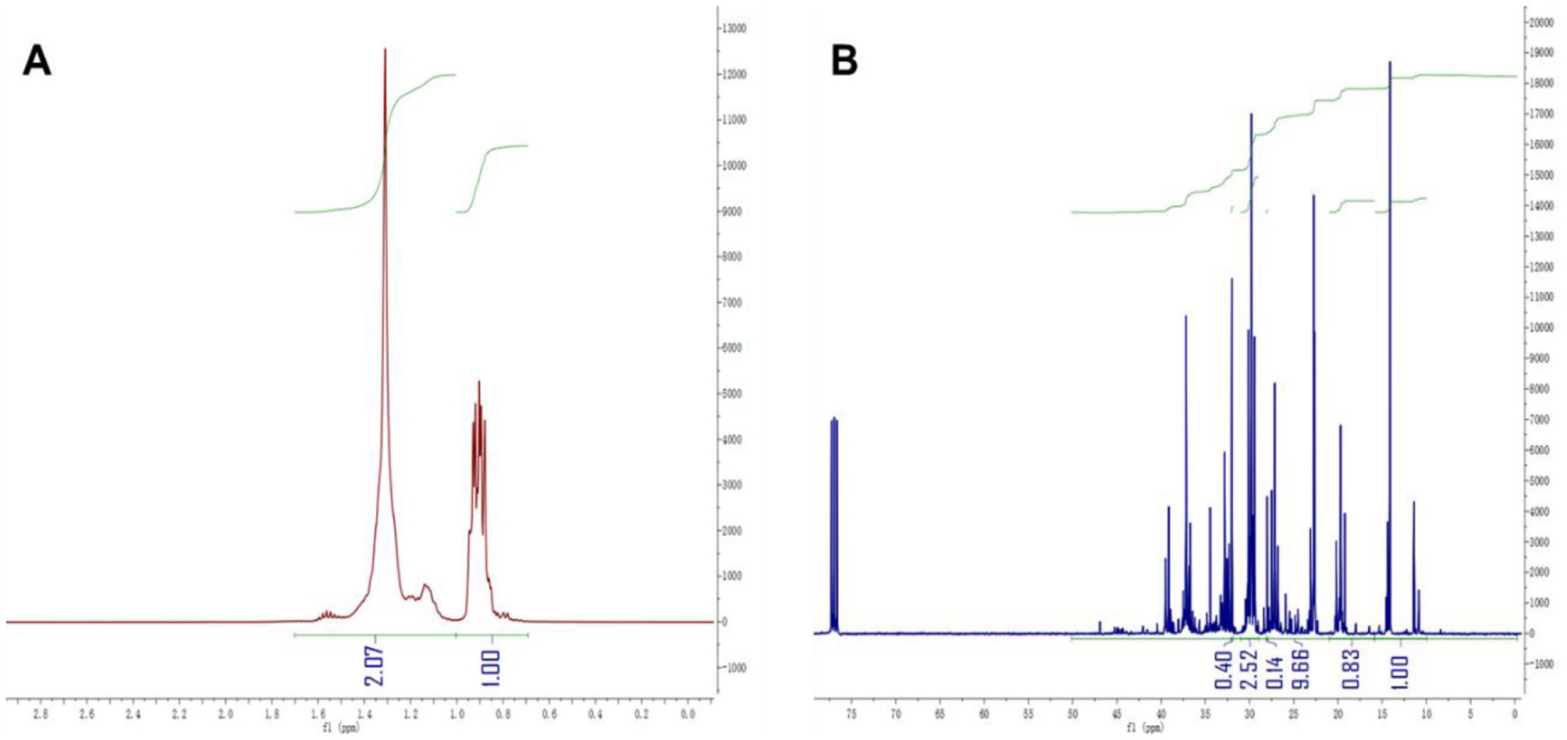
**(A)** N-15 ^1^H-NMR; **(B)** N-15 ^13^C-NMR (CDCl_3_).

**Figure 4:**
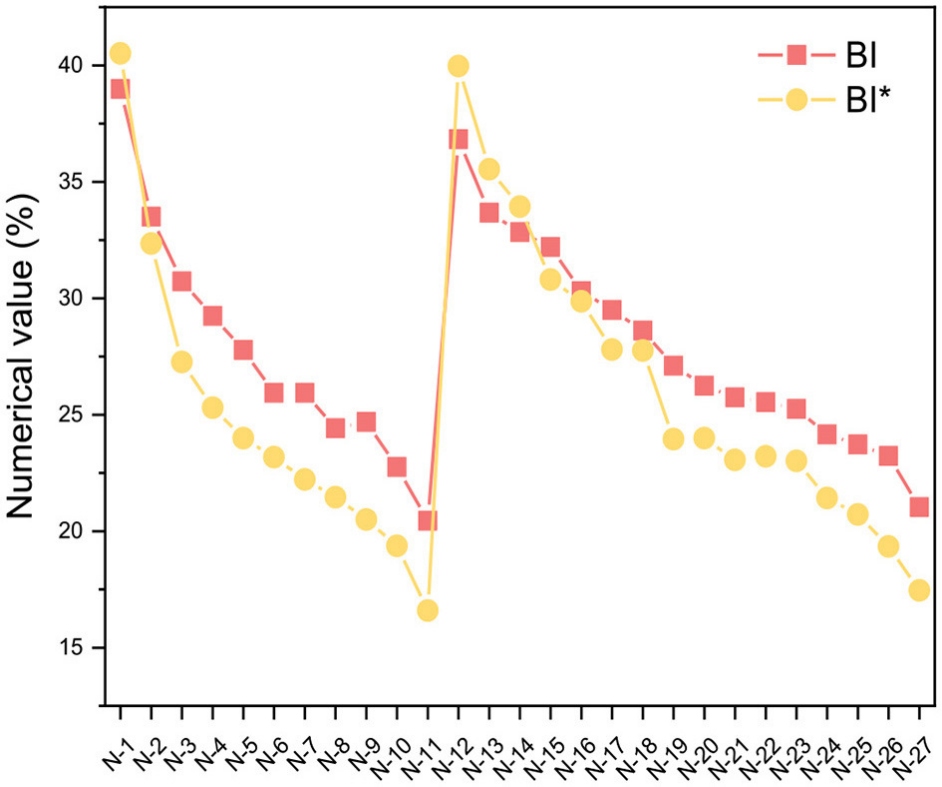
Comparison of ***BI*** and BI* calculations.

(4) Calculation example:

N-15 is a cut oil sample of FT lubricant base oil with a distillation range of 280–310°C, which belongs to the class analog oil. The N-15 is used as an example for the calculation:

The class analog oil N-15 was used as an example, and Equations 5–12 were employed for our structural parameter calculations. The integration results for each carbon and hydrogen spectral interval are shown in Figure 3, and the results of the parameter calculations are presented in Table 2. From the calculation results, it can be observed that the BI^*^ calculated by ^13^C-NMR is slightly smaller than the *BI* value calculated by ^1^H-NMR, which is consistent with the results from the model compounds in Table 1. In fact, we performed ***BI*** and BI^*^ calculations on all samples of class analog oils and found that there is an approximately equal relationship between the two, as shown in Figure 4. This suggests that ^13^C-NMR calculations can be used for structural calculations of FT lubricant base oils.

**Table 2:** Calculated structures using each structural parameter of equations.

**Number**	**tMe/%**	**bMe/%**	**NP/%**	**IP/%**	***C*** ^*^	***B***	***BI*** ^*^/%	***BI*** **/%**
N-15	10.35	10.04	36.65	54.97	17.70	1.94	30.81	32.21

### 3.2 Structural parameters and performance correlation of class analog oils

**Figure 5:**
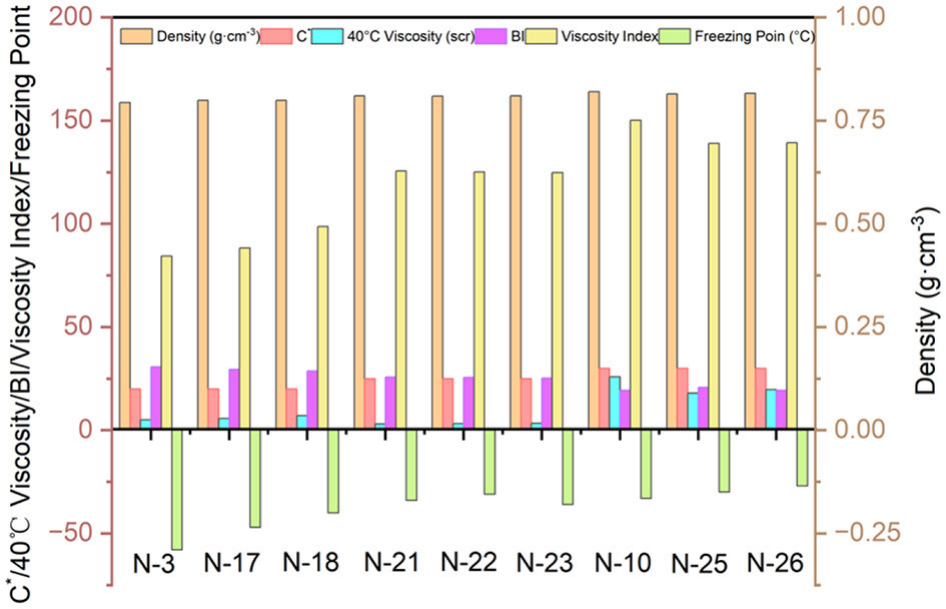
The relationship between structural parameters and physical-chemical properties.

We selected nine representative samples from a large number of analog-like oils to conduct a preliminary study of their constitutive relationships. As shown in Figure 5, it was found that as the ***BI*** increases, the density, viscosity, viscosity index, and condensation point decrease for approximately the same C^*^. For approximately the same ***BI***, the density, viscosity, viscosity index, and condensation point increase with increasing C^*^. It is evident that the two structural parameters, C^*^ and ***BI***, are closely related to the physicochemical properties of lubricant base oils. Therefore, it is essential to have an accurate understanding of their structural information. To further study the constitutive relationship between structure and performance, the structural parameters of the class analog oils were correlated with the four basic physicochemical properties, and empirical equations were initially derived. The structural information parameters and physicochemical properties of the other classes of simulated oils are presented in Supplementary Table S3.

#### 3.2.1 Fitting of structural parameters of class analog oil associations to oil properties

**Figure 6:**
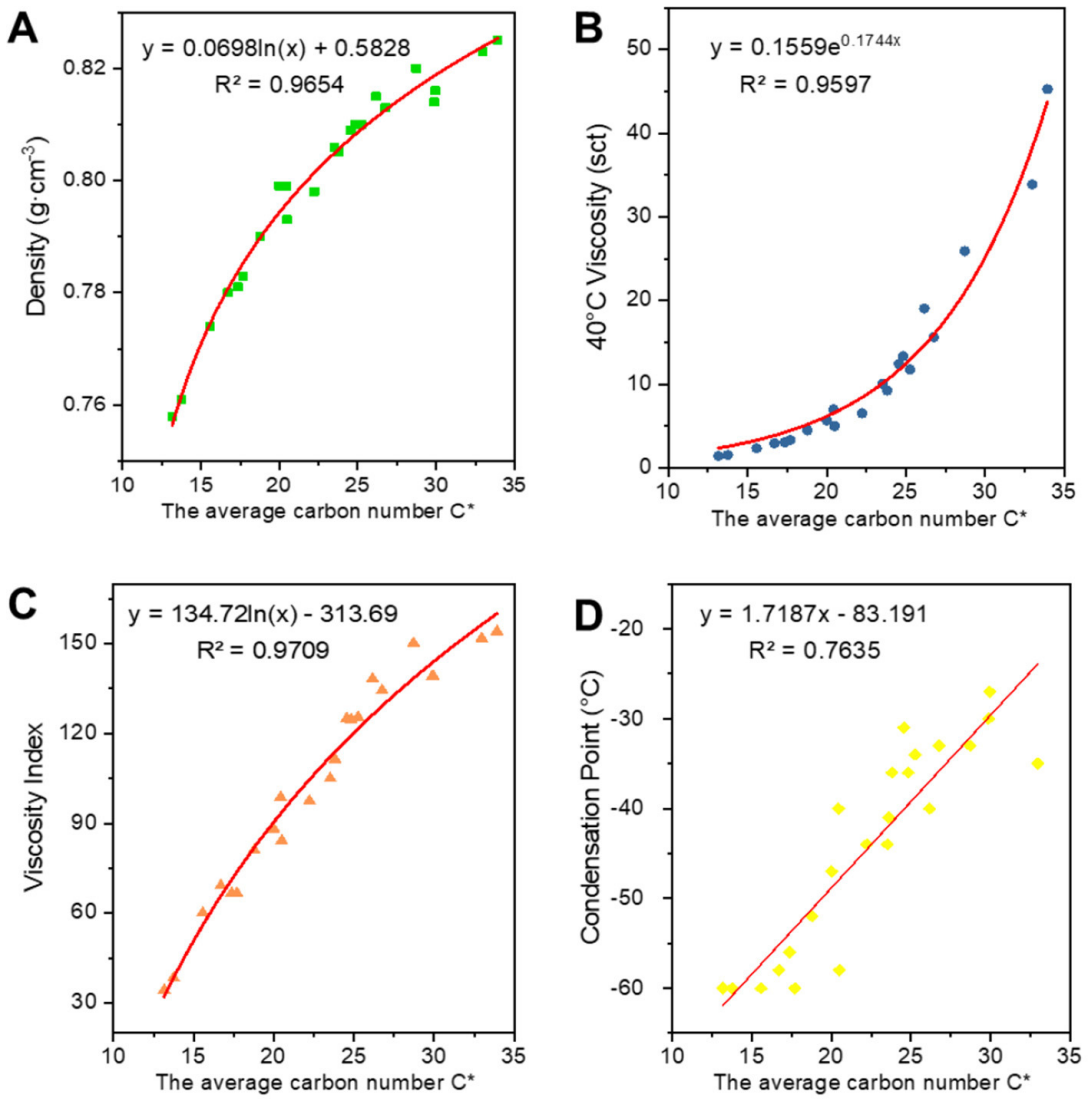
Correlation of C* with physico-chemical properties: **(A)** indicates association with density; **(B)** indicates correlation with 40°C viscosity; **(C)** indicates correlation with viscosity index; **(D)** indicates the correlation with condensation point, the same below.

**Figure 7:**
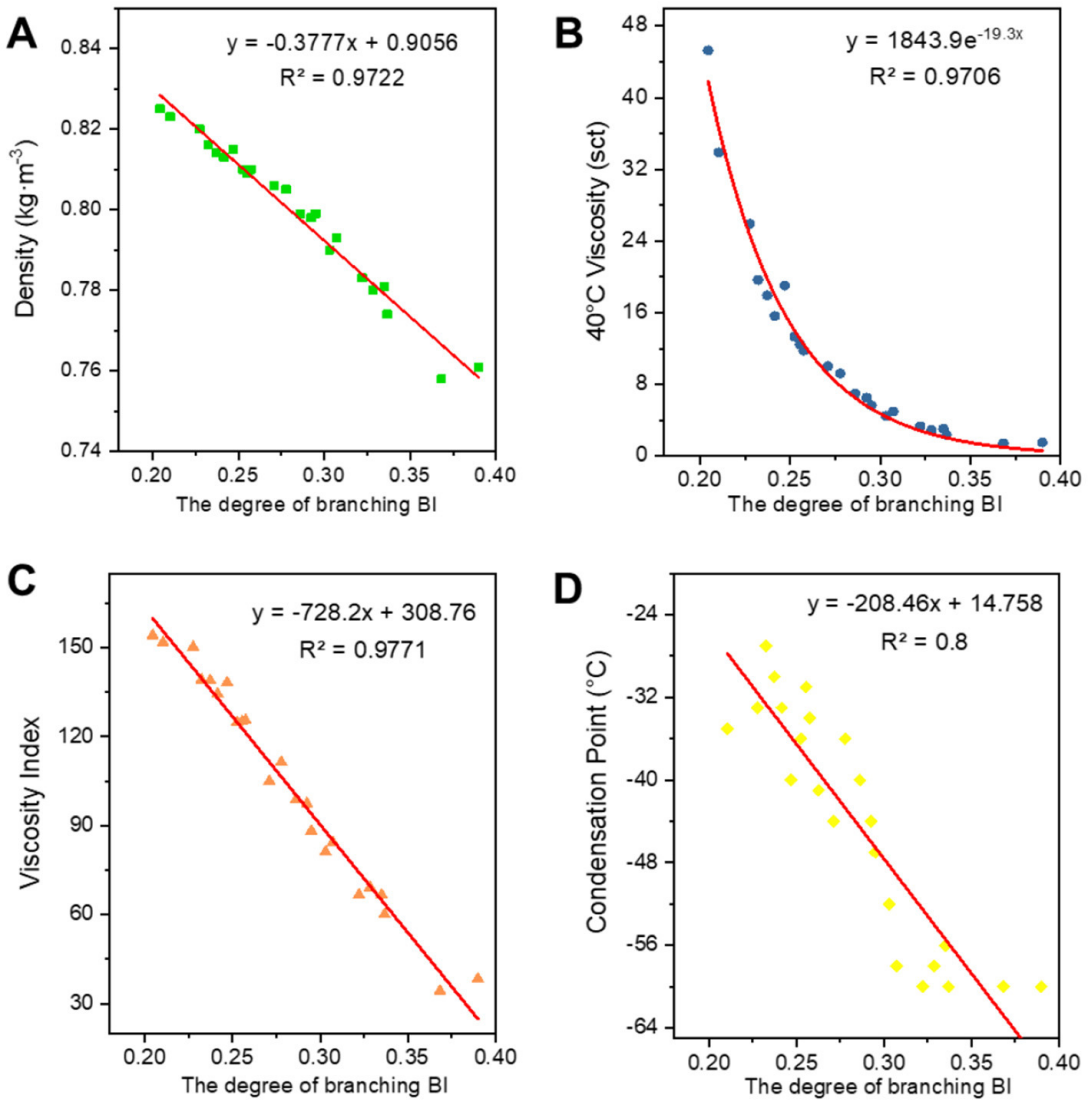
Correlation between ***BI*** and physicochemical properties. **(A)** Density; **(B)** 40°C viscosity; **(C)** viscosity index; and **(D)** condensation point.

**Figure 8:**
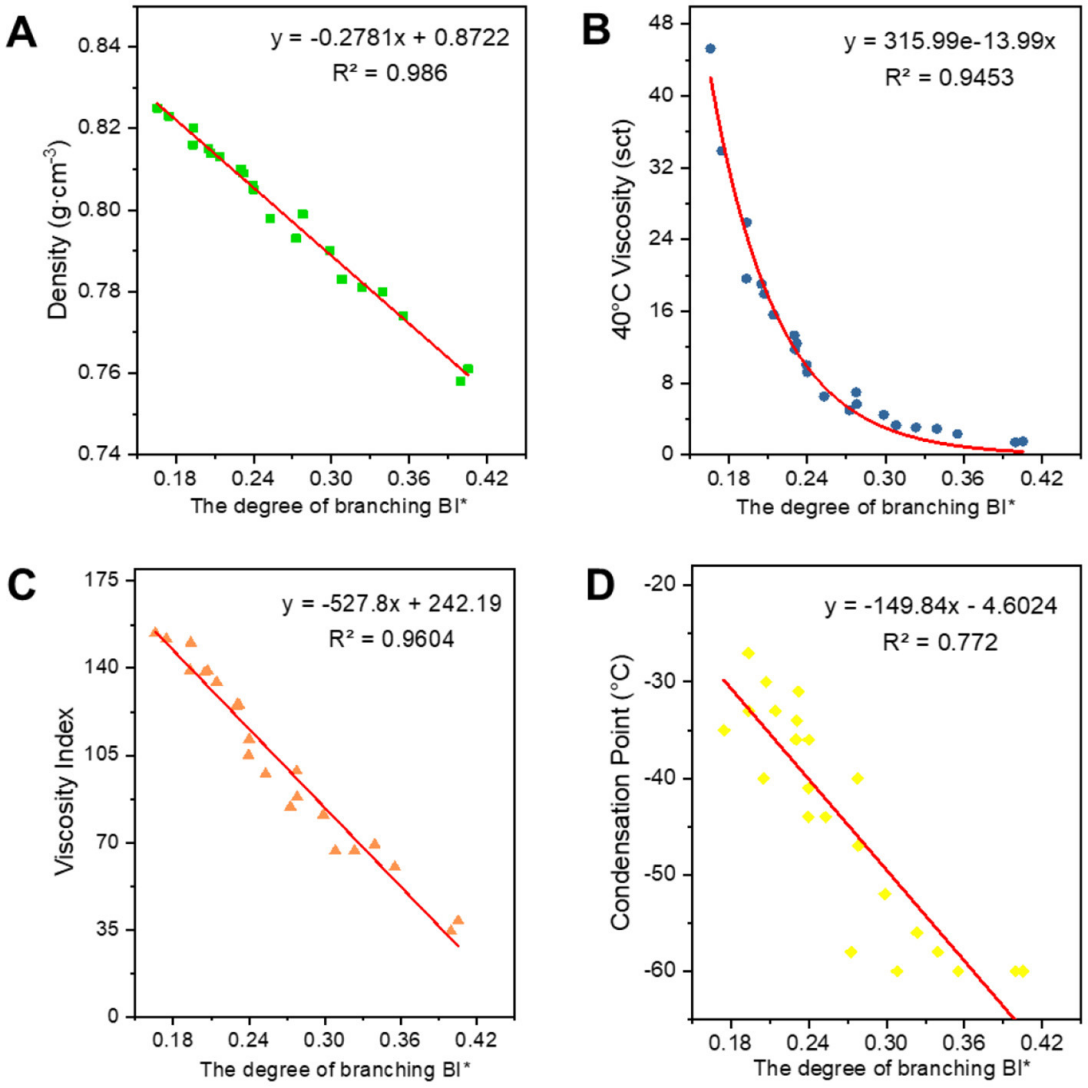
Correlation between BI* and physicochemical properties. **(A)** Density; **(B)** 40°C viscosity; **(C)** viscosity index; and **(D)** condensation point.

Figures 6–8 show the fitted data plots of the constitutive correlations of C^*^, BI, and BI^*^ with the density (a), 40°C viscosity (b), viscosity index (c), and condensation point (d) of the analog-like oils, respectively. From the fitted results, it is observed that there is a good linear or exponential relationship between each property and C^*^, ***BI***, and BI^*^.

There is a positive correlation between C^*^ and each of the attributes, with these attributes gradually increasing as C^*^ increases. Density primarily reflects changes in the distillation range and the average molecular weight of the base oil. The longer the average carbon chain, the higher the average molecular weight of the base oil, which results in a higher density. Viscosity is an index used to assess the fluidity of oils, indicating the extent of intermolecular frictional resistance during fluid movement. Therefore, the kinematic viscosity of base oil is closely related to the size and structure of the molecules in its composition. An increase in intermolecular frictional resistance between long-chain hydrocarbon components with a high average carbon number leads to an increase in viscosity. Viscosity index is an indicator that reflects the viscosity and temperature properties of base oils. The increase in the average carbon number of molecules is reflected in the macroscopic physical properties, such as the increase in boiling point, which leads to a higher viscosity index. The condensation point is a conventional indicator used to measure the low-temperature fluidity of lubricants. The longer the carbon chain, the stronger the intermolecular interaction between hydrocarbons, making it easier to form a crystalline structure that allows crystals to gradually precipitate out, resulting in a higher condensation point.

Regarding the correlation between ***BI*** and BI^*^ and various properties, density, viscosity, viscosity index (Wang et al., 2010), and condensation point are negatively correlated with them. The higher the degree of branching, the less favorable the growth of molecular chains, leading to a higher proportion of amorphous forms and a lower proportion of crystalline states, which results in a decrease in density. The bending of molecular chains tends to cause them to shrink into a group, reducing molecular entanglement and the dispersive forces between neighboring molecules, which in turn leads to a decrease in viscosity. The viscosity and temperature properties of isomeric alkanes with a smaller degree of branching are slightly worse than those of n-alkanes. As the degree of branching increases, the viscosity and temperature properties worsen. Additionally, the higher the degree of isomerization, the more irregular the molecular shape becomes, the greater the molecular gap, and the weaker the dispersive forces between neighboring molecules, resulting in a decrease in the condensation point. The average carbon number and degree of branching have opposing effects on the properties, ultimately indicating that these properties are the result of mutual interactions.

#### 3.2.2 Optimal structural parameters-BC^*^

**Figure 9:**
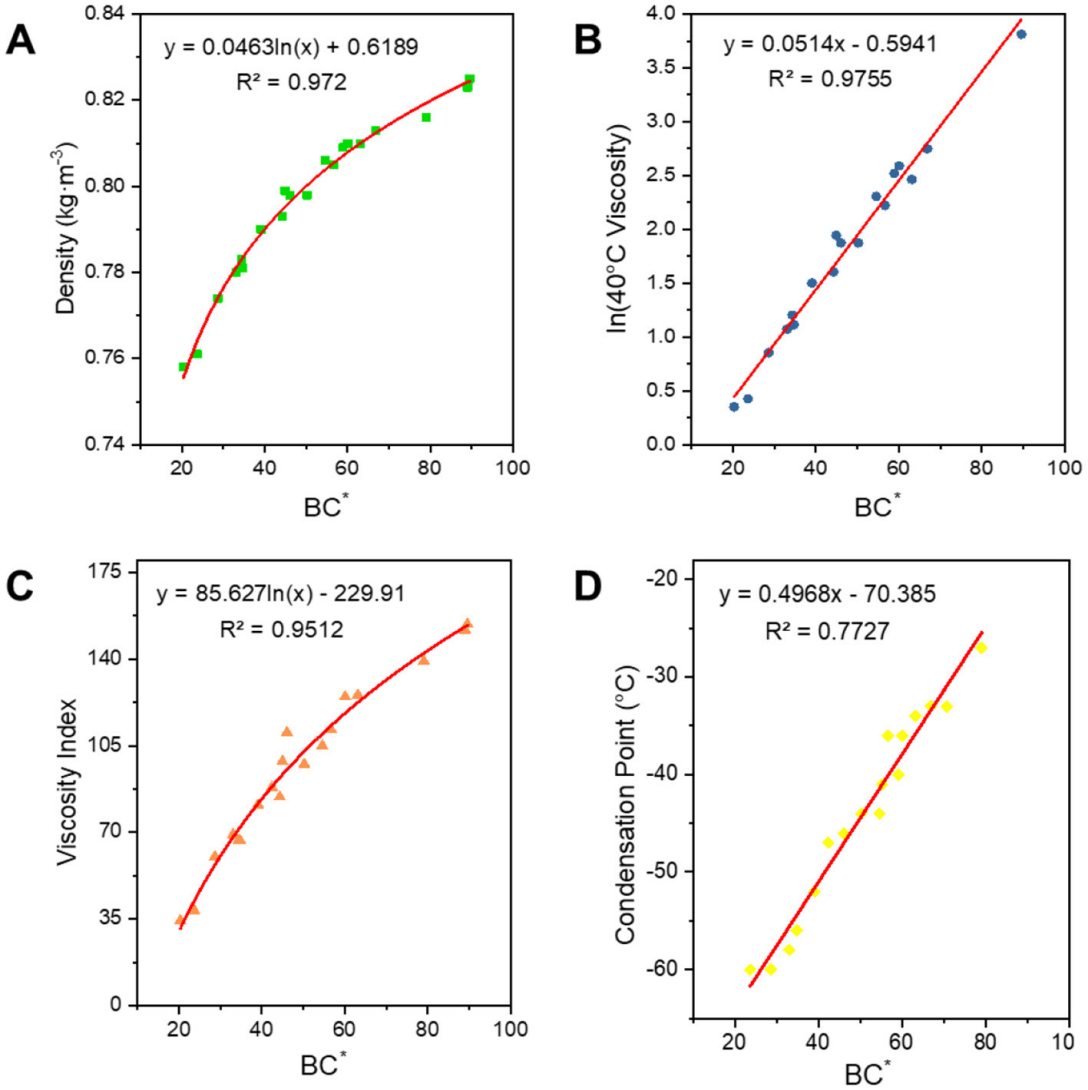
Correlation of structural indices with physicochemical properties. **(A)** Density; **(B)** 40°C viscosity; **(C)** viscosity index; and **(D)** condensation point.

The average carbon number is positively correlated with the properties, while the degree of branching is negatively correlated. To better express the combined influence of these two factors on the structural composition, we introduced a structural index, BC^*^. B represents the number of branched nodes, which is calculated using Equation 11 and reflects the average number of branched chains in the oil, providing an indication of the molecular structure's branching. To better understand and calculate this, we created the structural index BC^*^. As shown in Figure 9, its correlation with the properties of the oils was fitted, and empirical equations for the corresponding properties were derived. As can be seen from Table 3, all properties correlate well with the average carbon number C^*^ and the degree of branching ***BI***. The structural index BC^*^, which is derived from the correlation between the average carbon number and the number of branched chain nodes, also exhibits a strong correlation with the performance. Except for the condensation point, the fitting coefficients *R*^2^ for all properties were >0.95. BC^*^ can express both the carbon number and the branched chains simultaneously. Therefore, we initially selected BC^*^ as a structural parameter to correlate the physicochemical properties of the oil. From the correlation coefficient for the condensation point, we can see that the correlation between ***BI*** and BC^*^ is relatively low. The poor correlation for the condensation point is attributed to two factors: (1) the condensation point of FT lubricant base oils is too low (< −60°C) for accurate measurement by the instrument, and (2) the solidification process of long-chain alkanes involves mutual solubilization and coagulation effects, making the relationship between the condensation point and the structure more complex.

**Table 3:** Expression for fitting the structural relationship of the class analog oil.

**Properties**	***C*** ^*^	***BI***	***BI*** ^*^	***BC*** ^*^
Densities	*y* = 0.0698ln(*x*) + 0.5828 *R*^2^ = 0.9654	*y* = −0.3777*x* + 0.9056 *R*^2^ = 0.9722	*y* = −0.2781*x* + 0.8722 *R*^2^ = 0.986	*y* = 0.0463ln(*x*) + 0.6189 *R*^2^ = 0.972
40°C viscosity	*y* = 0.1559e^0.1744x^ *R*^2^ = 0.9597	*y* = 1843.9e^−19.3*x*^ *R*^2^ = 0.9706	*y* = 315.99e^−13.99*x*^ *R*^2^ = 0.9453	ln *y* = 0.0514*x* – 0.5941 *R*^2^ = 0.9755
Viscosity index	*y* = 134.72ln(*x*) – 313.69 *R*^2^ = 0.9709	*y* = −728.2*x* + 308.76 *R*^2^ = 0.9771	*y* = −527.8*x* + 242.19 *R*^2^ = 0.9604	*y* = 85.627ln(*x*) – 229.91 *R*^2^ = 0.9512
Condensation point	*y* = 1.7187*x* – 83.191 *R*^2^ = 0.7635	*y* = −208.46*x* + 14.758 *R*^2^ = 0.8	*y* = −149.84*x* – 4.6024 *R*^2^ = 0.772	*y* = 0.4968*x* – 70.385 *R*^2^ = 0.7727

#### 3.2.3 Class analog oil formula corroboration

**Figure 10:**
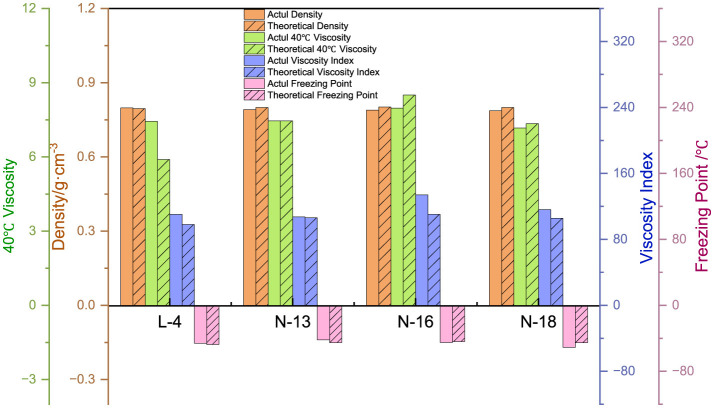
Confirmation of the expression for the constitutive fit of the analog-like oils.

The error between the calculated and measured values of each property, based on the class analog oil and BC^*^ fitted formulae, is shown in Figure 10 to corroborate the feasibility of our formulas. After selecting the narrow fraction samples and applying them to the empirical formula for calculation, it was found that their densities are very close to each other, with an error of < 2%. The errors in the 40°C viscosity and coagulation point of the sample oil are approximately 10%. The error in the viscosity index is larger, which is attributed to the volatilization of light components when the viscosity is measured at 100°C. It can be inferred that BC^*^ is a more efficient parameter to correlate structure and performance than other parameters, which will be applied to the establishment of FT lubricant base oils constitutive relationship in the following.

### 3.3 FT lubricant base oils constitutive relationship establishment

#### 3.3.1 Structural parameter correlation

FT lubricant base oils are a combination of analogue oils with a relatively long distillation range, and are therefore also known as wide-distillation oils. The expressive power of the average carbon number is discounted for wide fractions due to the fact that it is one of the fractions with a wide width span. Moreover, the inclusion of long-chain hydrocarbon carbons and branched chains is complex and therefore cannot be directly calculated by applying the formulae introduced by the analogue oils. It is necessary to derive the relevant constitutive relationship equations based on the nature of the wide-distillate oils with reference to the relationships of the class of model oils. The relational formula is in Table 4. Wide fractions have a wider carbon number distribution due to their longer boiling ranges, and the structural index and degree of branching do not correlate as well with the physical properties of the oil as narrow fractions.

**Table 4:** Fitted expressions for the conformational relationships of the broad fractions.

**Properties**	BC^*^	BBC^*^
Densities	*y* = 0.11ln(*x*) + 0.2583 *R*^2^ = 0.9204	*y* = 0.0001*x* + 0.7187 *R*^2^ = 0.9832
ln(40°C viscosity)	*y* = 0.0306*x* – 1.8789 *R*^2^ = 0.9246	*y* = 0.006*x* – 1.4258 *R*^2^ = 0.9579
Viscosity index	*y* = −1.9742*x* + 431.26 *R*^2^ = 0.9122	*y* = −0.3086*x* + 344.62 *R*^2^ = 0.9197
Condensation point	*y* = 0.2837*x* – 81.562 *R*^2^ = 0.7583	*y* = 0.0482*x* – 72.133 *R*^2^ = 0.8142

**Figure 11:**
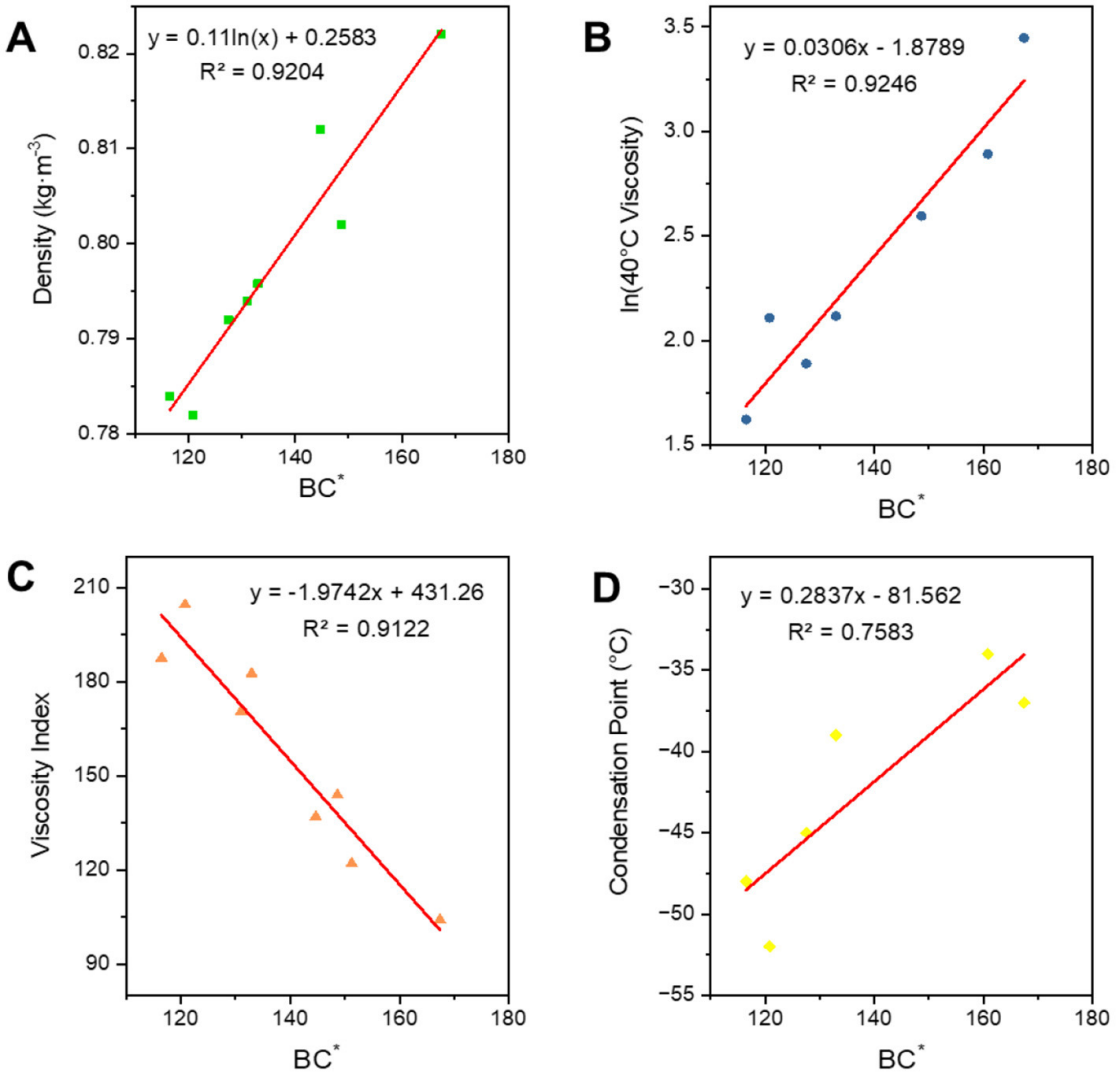
Correlation of FT lubricant base oil structure indices. **(A)** Density; **(B)** 40^*^C viscosity; **(C)** viscosity index; and **(D)** condensation point.

**Figure 12:**
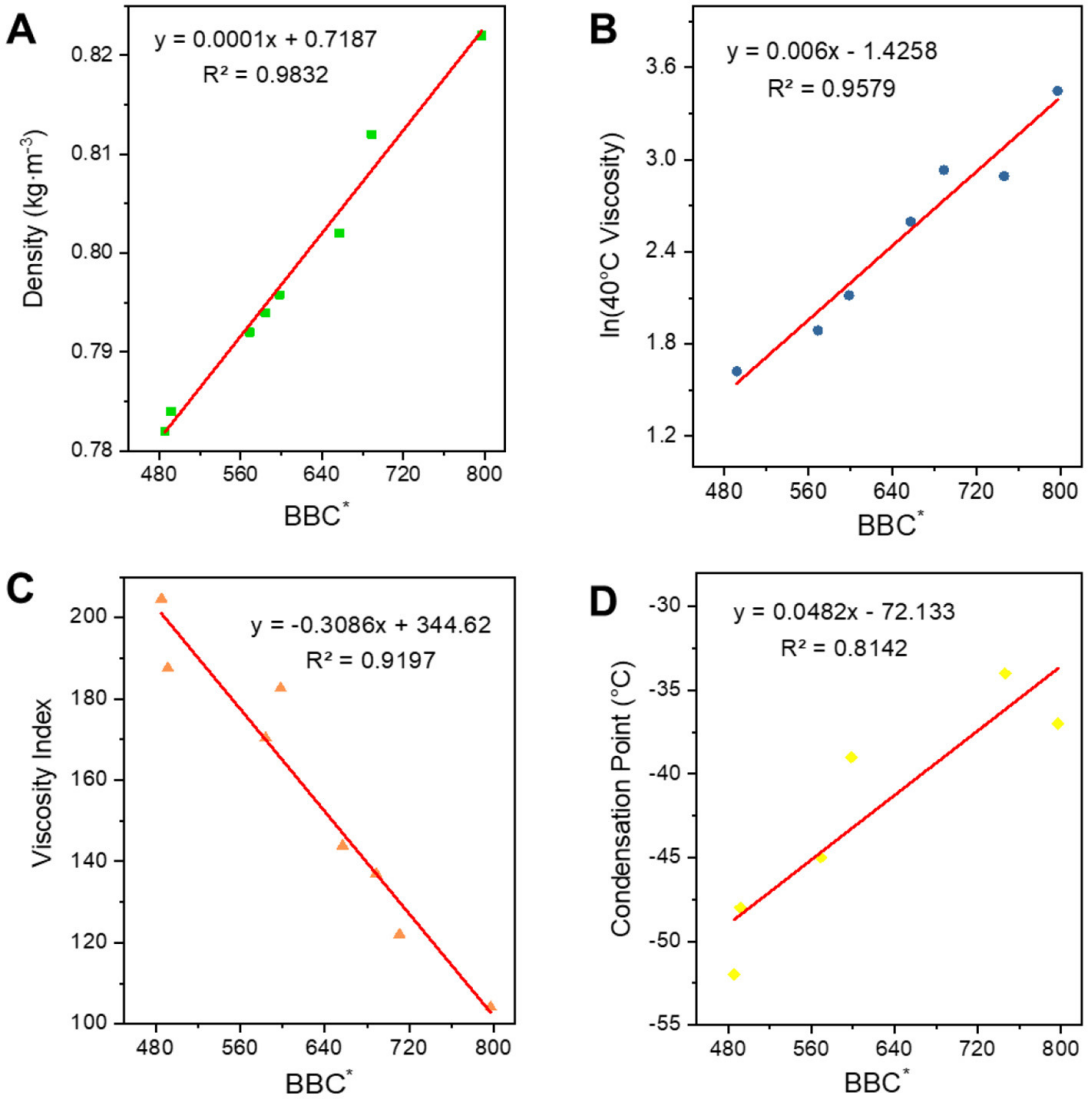
Correlation of BBC* with physicochemical properties. **(A)** Density; **(B)** 40^*^C viscosity; **(C)** viscosity index; and **(D)** condensation point.

However, as can be seen in Figure 11 their trends are consistent, and the *R*^2^ of the fit between BC^*^ and the four properties is less than 0.95, which is not a very good correlation. So, we added the parameter ***B*** which can express the width of the carbon number to correct to get a new parameter BBC^*^, and then carried out the correlation of the constitutive relation as shown in Figure 12. In terms of the fitting coefficient *R*^2^, BBC^*^ is more effective than ***BC***, and it's fitting formula is closer to the results of the predictive nature. The structural information and physicochemical properties of FT lubricant base oils can be found in Supplementary Table S4. BBC^*^ shows a stronger correlation than BC^*^, and its fitting formula is closer to the predicted property results.

#### 3.3.2 Corroborate by means of a formula

**Figure 13:**
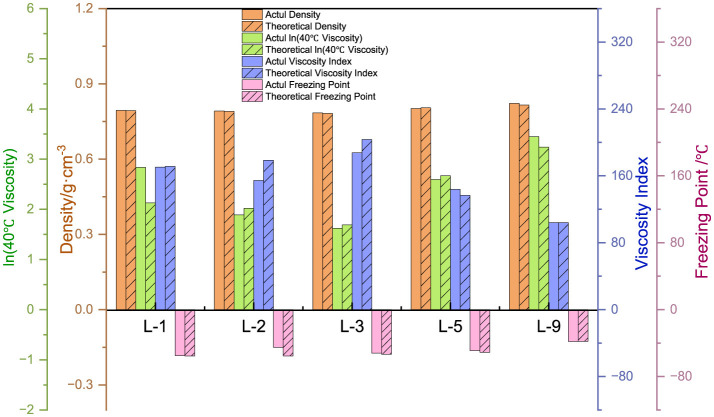
FT lubricant base oil constitutive fit expression corroboration.

Based on the properties of FT lubricant base oil and the BBC^*^ fitting formula, the attribute values are calculated. The error is then compared with the measured values to confirm the feasibility of the formula. As shown in Figure 13, the calculated density is very close to the actual density, with an error of < 3%. Similarly, the calculated viscosity at 40°C is also close to the actual value, with an error of < 5%. The error in the viscosity index is larger, within 10%, which is related to the volatilization of light components when measuring viscosity at 100°C during the test. The error in the condensation point is also relatively large, within 30%. This is partially attributed to the testing instrument and human error in measurement. Overall, it is possible to predict the performance of FT lubricant base oils to a certain extent.

### 3.4 Physical and chemical property correlation

There is a direct relationship between the class analog oil and the FT lubricant base oil, meaning that the simulated oil is a component extracted from the FT lubricant base oils based on different temperature ranges. Specifically, N-1–N-11 are narrow fractions cut from L-7 based on different fractions, while N-13–N-27 are narrow fractions cut from L-3 based on different fractions. We calculated the percentage content of each narrow fraction by dividing the carbon number distribution into intervals, then applied the density fitting formulas for *BI* and BC^*^, respectively. The calculated density structure is shown in Table 5. The densities calculated using the above two fitting formulas are very close to the actual densities, confirming the feasibility of the formulas. The empirical formula for BC^*^ from Table 3 was tested on another 40 blended oil samples produced under different catalysts and process conditions, with the results shown in Table 6. It can be seen that the formula is accurate and has a wide range of applicability.

**Table 5:** **BI** and BC^*^ association density calculation data sheet.

**Oil designator**	**Density/g.cm^−3^**	***BI*** **fitting formula to calculate density/g.cm^−3^**	BC^*^fitting formula to calculate density/g.cm^−3^
L-3	0.7950	0.8001	0.7993
L-7	0.7880	0.8073	0.8083

**Table 6:** Data calculation table (the formula from Table 3 BC^*^).

**No. oil**	**Structure index**	**Calculated density/g/cm^3^**	**Density/g/cm^3^**	**Calculated viscosity/mm^2^·s^−1^**	**Viscosity at 40°C/mm^2^·s^−1^**	**Calculated viscosity index**	**Viscosity index**	**Calculated condensation point/°C**	**Condensation point/°C**
1	61.39	0.801	0.7915	6.2	7.8	122.6	135	−40	−52
2	56.26	0.797	0.7907	5.7	7.8	115.2	107	−42	−49
3	51.51	0.793	0.7922	5.2	7.8	107.6	107	−45	−51
4	81.23	0.814	0.7937	8.2	7.9	146.6	158	−30	−52
5	81.65	0.814	0.8064	8.3	8.1	147.1	146	−30	−54
6	50.64	0.792	0.7915	5.1	7.5	106.1	107.3	−45	−42
7	52.65	0.794	0.7958	5.3	8.4	109.5	139.2	−44	−27
8	53.19	0.794	0.8043	5.4	6.7	110.4	96.0	−44	−45
9	50.34	0.791	0.7871	5.1	7.2	105.6	116.0	−45	−51
10	45.16	0.786	0.7943	6.2	5.7	96.3	88.2	−48	−47

Mineral oils are cost-effective and have a wide range of applications, but they suffer from poor thermal stability, oxidative stability, and low-temperature fluidity, and contain higher levels of impurities. Among these impurities, nitrogen, sulfur, and aromatic compounds not only negatively affect the environment but also significantly impact the performance of the base oil. FT lubricant base oil, on the other hand, consists of a single component—long-chain alkanes—offering good low-temperature fluidity, excellent oxidation resistance, and a viscosity index slightly lower than that of PAO base oils. PAO base oils exhibit extremely low volatility, good low-temperature fluidity, and long service life, but their cost is significantly higher than that of FT lubricant base oils. In summary, FT lubricant base oils are composed solely of long-chain alkanes, offering excellent low-temperature fluidity, good oxidation stability, and a viscosity index slightly lower than that of PAO base oils, which are characterized by very low volatility, good low-temperature fluidity, and extended service life, albeit at a much higher price than FT lubricant base oils.

Subsequently, we reviewed the relevant literature and organized several common Fischer-Tropsch oils (Liu et al., 2024), PAO (Xue et al., 2021; Porfiryev et al., 2020), and mineral oil base oils (Porfiryev et al., 2020; Gross, 2022). Based on the structural parameters and physical property data provided in the literature, we applied the obtained structural parameters to the calculation formulas in Table 3 BC^*^ from our article. The results of the calculations are shown in Table 7. From the table, it can be observed that our formula has good applicability for straight-chain alkanes, such as Fischer-Tropsch oils and PAO. However, it does not fit well for oils containing aromatic hydrocarbons, such as oil S-9 (Gross, 2022), and the fitting results are not informative.

**Table 7:** Comparative performance data.

**Type of oil**	**Oil name**	**Structure index**	**Calculated viscosity/mm^2^·s^−1^**	**Viscosity at 40°C/mm^2^·s^−1^**	**Calculated viscosity index**	**Viscosity index**	**Calculated condensation point/°C**	**Condensation point/°C**
F-T oil (Liu et al., 2024)	CTL4	108	5.6	3.94	121.7	128	−16	−33
	YU4	100	5.2	4.12	115.3	119	−20	−21
	GTL4	89	4.6	4.063	155	121	−26	−39
PAO (Xue et al., 2021)	Mpao4	110	5.7	3.85	173.1	125	−55	−75
	PAO4–M	75	3.9	4.12	140.2	122	−73	−66
	Trimer 1-octene	48	2.5	2.05	101.6	111	−87	−75
	Tetramer 1-octene	78	4	3.56	143.1	123	−72	−57
	Trimer 1-decene	56	2.9	0.58	114.8	142	−83	−66
	Tetramer 1-decene	90	4.6	5.94	155.4	146	−66	−54
	Trimer 1-dodecene	64	3.3	4.67	126.2	150	−79	−55
	PAO-2	45	2.3	1.9	96	–	−88	−70
	PAO-4	65	3.3	4.06	127.5	124	−78	−68

## 4 Conclusions

NMR can be effectively used to analyze the structural composition of lubricant base oils. When combined with high-temperature gas-phase assisted calculation methods, it is also possible to obtain more detailed structural information on the distillate, such as C^*^, ***BI***, ***B***, BC^*^, and BBC^*^. This information helps to provide a clearer understanding of the structure of the blended oils, facilitating a more accurate study of conformational relationships. A large amount of data has been used to establish constitutive relationships between the physicochemical properties of lubricant base oils and key structural parameters. The density, condensation point, viscosity, and viscosity index of base oils are positively correlated with C^*^ and negatively correlated with ***BI*** (BI^*^). The effective structural parameters BC^*^ and property fitting equations were initially derived through analog oil correlation calculations. The *R*^2^ value was as high as 0.95 or higher, except for the condensation point. Subsequently, these equations were applied to calculate the compositional relationships of FT oils, yielding good results. Except for the condensation point, the errors were within 10%, confirming the feasibility of the formula.

## Data Availability

The original contributions presented in the study are included in the article/Supplementary material, further inquiries can be directed to the corresponding author.
