# Supplementary material for: Using nuclear magnetic resonance to assist in calculating the structure of Fischer-Tropsch lubricant
Source: Front Res Metr Anal. 2025 May 1;10:1415831. doi: 10.3389/frma.2025.1415831 (PMC12078291; doi:10.3389/frma.2025.1415831)
Supplement: Supplementary file 1 [file Data_Sheet_1.docx]

Using nuclear magnetic resonance to assist in calculating the structure of Fischer-Tropsch lubricant

Supplementary Material

**1.** **Influence of the position of the branched chain**

Table S1 Chemical shift diagrams for different branch positions

| No. | Carbon skeleton structure and position of characteristic peaks | chemical shift radiation |
| --- | --- | --- |
| S1 | 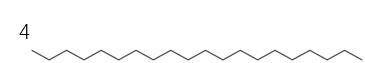 | 14.17 |
| S2 | 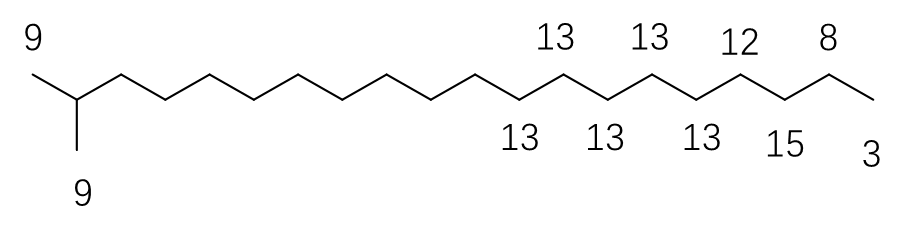 | 23.10 |
| S3 | 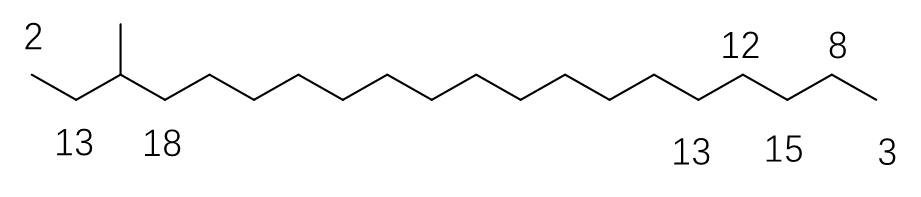 | 11.4 |
| S4 | 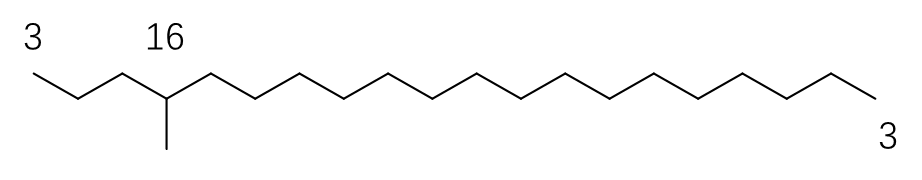 | 32.79 |
| S5 | 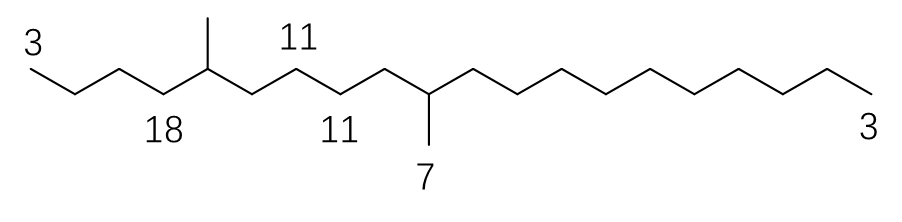 | 27.48 |
| S6 | 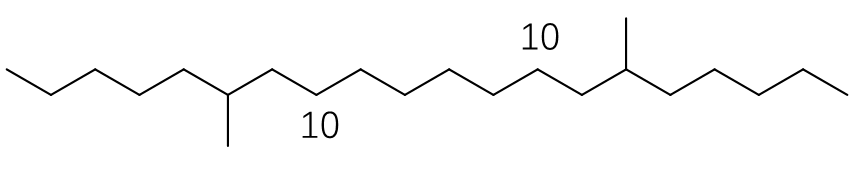 | 27.17 |
| S7 | 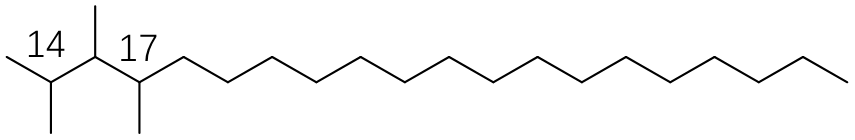 | 34.43 |
| S8 | 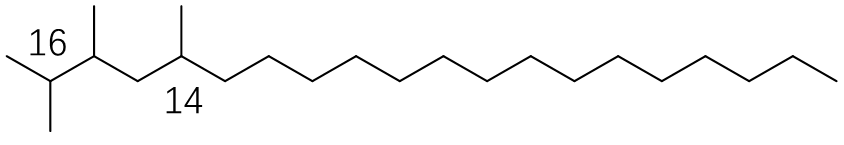 | 30.09 |
| S9 | 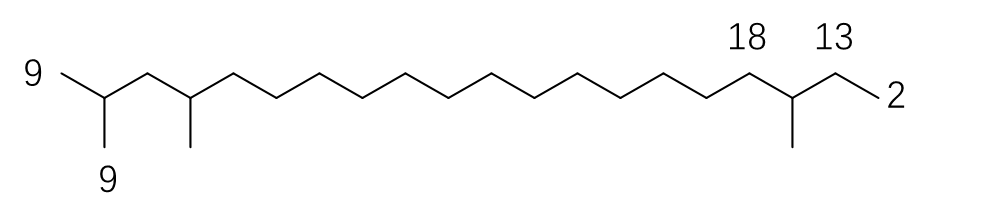 | 37.09 |
| S10 | 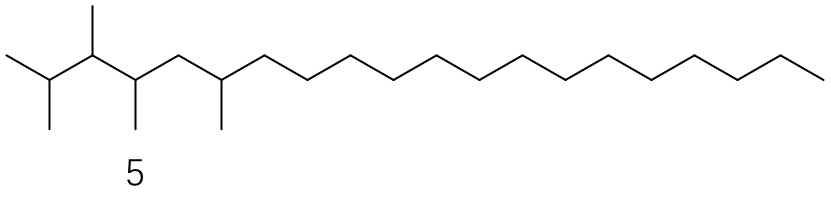 | 19.25 |

From Table S1, it can be seen that for the model compounds, the substituent characteristic peaks can be used to judge the branched position S. For the FT lubricant base oils, which are mixed substances, there are several branched positions S in the structure, and the characteristic peaks cover each other, so that the differentiation cannot be achieved, and therefore the effect of the branched position S cannot be accurately presented.

**2. Analysis and Division of NMR Spectra**


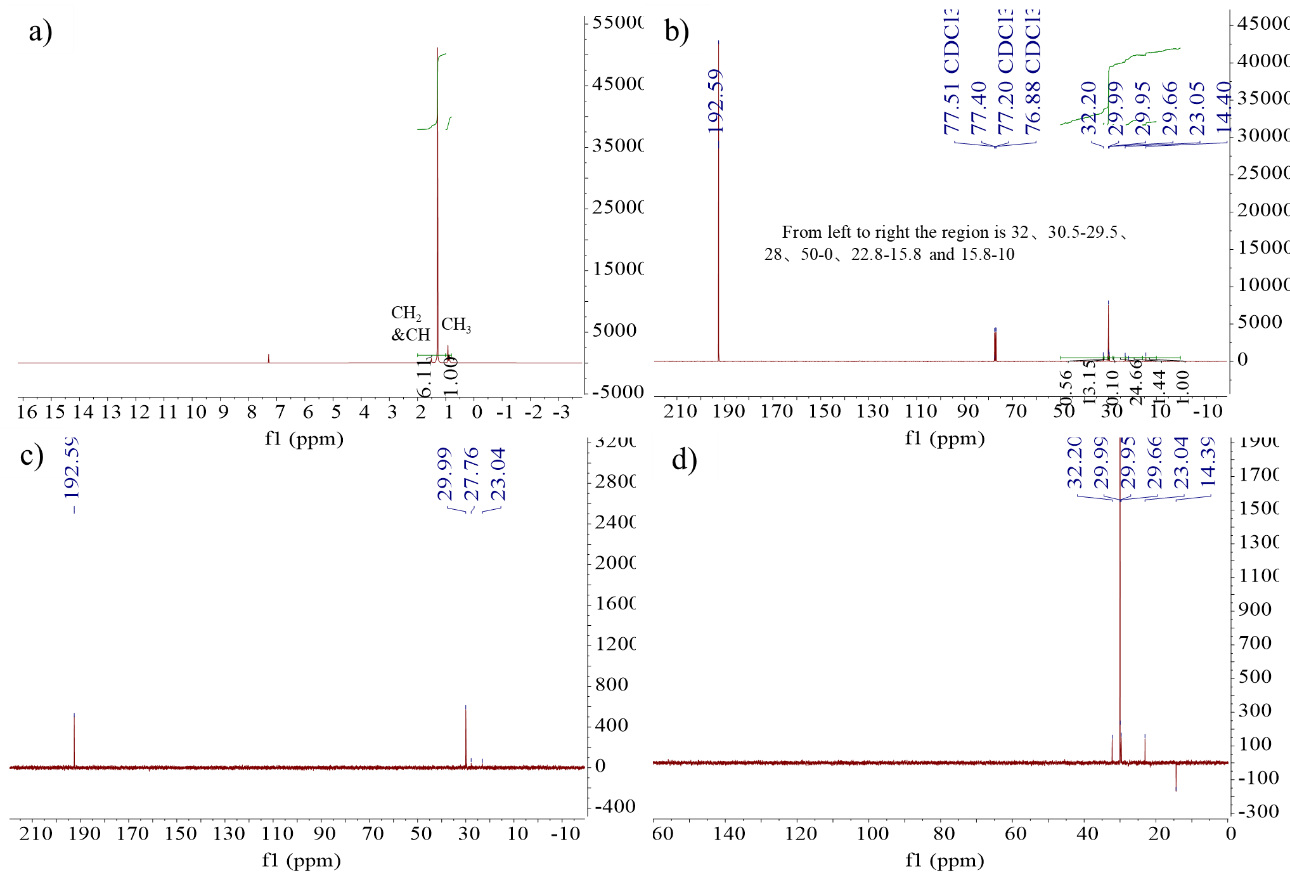


Figure S1 ^1^H-NMR spectrum (a), ^13^C-NMR spectrum (b), 90° DEPT spectrum (c), and 135° DEPT spectrum (d) of L-1

Table S2 Characteristic Chemical Shift Ranges of Long-Chain Alkanes Simulated by ChemDraw

|  |  | Simulated chemical shift | Chemical shift range |
| --- | --- | --- | --- |
| ^1^H-NMR | Primary hydrogen | 0.83, 0.86, 0.87, 0.88, 0.89, 0.91, 0.99 | 0.8-1.0 |
|  | Secondary hydrogen | 1.13, 1.19, 1.25, 1.26, 1.29, 1.31, 1.55 | 1.1-1.3, 1.55 |
|  | Tertiary hydrogen | 1.62, 1.40, 1.41 | 1.4, 1.62 |
| ^13^C-NMR | Primary carbon | Terminal methyl ：11.6, 14.1, 23.2, 23.5  Branched methyl：16.0, 16.3, 18.8, 21.3, 20.7, 21.0, 21.3, 21.6 | Terminal methyl: 10-15.8, 23.2  Branched methyl: 15.8-23.0 |
|  | ？Secondary carbon | 22.7, 23.0, 26.8, 27.1, 29.3, 29.6, 29.7, 29.9, 31.9, 32.1 34.6, 34.9, 35.2, 37.1, 37.4, 39.6, 42.1, 44.6, 45.5, 47.1, 47.4 | 29.2-30.7,  37.2 |
|  | Tertiary carbon | 25.6, 27.9, 28.1, 28.2, 28.4, 30.4, 31.0, 32.5, 32.9, 33.5, 35.4, 35.7, 36.8, 39.7, 42.2 | 28.0,  32.0 |

The samples were shaken and shaken well to take about 0.5 mL of the samples with a dropper, and the samples were tested by ^1^H-NMR, ^13^C-NMR, 90°dept spectra and 135°dept spectra using a Bruker Ascend nuclear magnetic resonance spectrometer at 400 M, using deuterated chloroform as the solvent. As shown in Figure S1 (a), the chemical shift range of primary hydrogen is 1.0-0.8 ppm with an integral value of 1. The chemical shift range of secondary hydrogen and tertiary hydrogen is 2.0-1.0 ppm with an integral value of 6.67. In the 135° dept spectrum, the methyl and isopropyl peaks are positive, while the methylene peak is negative. In the 90° spectrum, only the isopropyl peak is visible. Quaternary carbons do not show any signals in all dept spectrum, hence their peak positions can be determined by subtracting from the ^13^C-NMR spectrum. By comparing Figure S1 (c) and Figure S1 (d) the peak at 14.39 ppm is identified as the terminal methyl peak, the peaks at 30-29 ppm are identified as the methylene peaks, and the peaks at 32 ppm and 28 ppm are identified as the isopropyl peaks**.** Figure S1 (b) is compared with the dept spectrum and simulation results to determine that the peak at 14.39 ppm is the terminal methyl peak, the peaks at 23.05 ppm and 19.91 ppm are the branched methyl peaks, and the peak at 37 ppm is the quaternary carbon peak. By allocating the peaks in the spectrum, the specific chemical shift regions for different types of carbons can be characterized, which are used to determine the range of integrals in the calculation formulas for structural parameters.

Based on the ^13^C-NMR, ^1^H-NMR, DEPT spectrum and extensive ChemDraw simulation, the structural details and chemical shift values were attributed. The result of simulated chemical shifts is listed in Table S2.

**3.** **Statement of reasonableness of calculations**

Based on the inherent structural characteristics of FT lubricant base oil, the formulas for structural parameters and the range of chemical shifts have been refined This refinement ensures that the formulas are more targeted and capable of accurately calculating the structural parameters of FT-based lubricant base oil. Taking the average carbon number (C*) as an example, as depicted in Figure S2, simulations distillation, elemental analysis, and NMR can all be employed to compute the average molecular weight of FT oil. However, the individual accuracy of each method is insufficient. By conducting a comparative analysis and cross-validating the results obtained from these three methods, more reliable outcomes can be attained, thereby demonstrating the feasibility of the derived NMR calculation formulas.

Figure S2 Comparison of average chain lengths obtained through simulated distillation, elemental analysis, and NMR calculations**.**

**4.** **Structural information and physical and chemical properties of various oils**

The structural information of the analogue and FT lubricant base oils calculated based on Eqs. 9-12, as well as the density, 40°C viscosity, viscosity index and freezing point physicochemical properties are shown in Table S3 and Table S4. Class analogue oils is used for system research, to find the relevant structural parameters, and to establish the structure-activity relationship. The FT lubricant base oil is a real oil and is used to construct structure-activity relationships and verify fitting equations.

Table S3 Data Sheet on Structural Parameters and Physicochemical Properties of Class Analogue Oil Types

| No. | C* | BI/% | B | BI*/% | Density /g.cm^-3^ | 40℃ viscosity /mm^2^.s^-1^ | Viscosity index | Condensation point,/℃ |
| --- | --- | --- | --- | --- | --- | --- | --- | --- |
| N-1 | 13.76 | 38.99 | 1.72 | 40.52 | 0.761 | 1.53 | 38.4 | -60 |
| N-2 | 17.37 | 33.50 | 2.00 | 32.34 | 0.781 | 3.05 | 66.7 | -56 |
| N-3 | 20.50 | 30.72 | 2.16 | 27.26 | 0.793 | 4.98 | 84.3 | -58 |
| N-4 | 22.23 | 29.24 | 2.26 | 25.30 | 0.798 | 6.52 | 97.5 | -44 |
| N-5 | 23.82 | 27.78 | 2.38 | 24.00 | 0.805 | 9.23 | 111.4 | -36 |
| N-6 | 25.40 | 25.94 | 2.54 | 23.18 | ˗ | - | - | - |
| N-7 | 25.89 | 25.94 | 2.48 | 22.21 | - | - | - | - |
| N-8 | 26.97 | 24.42 | 2.54 | 21.46 | - | - | - | - |
| N-9 | 26.17 | 24.69 | 2.26 | 20.50 | 0.815 | 19.03 | 138.3 | -40 |
| N-10 | 28.72 | 22.75 | 2.46 | 19.37 | 0.82 | 25.91 | 150.2 | -33 |
| N-11 | 33.93 | 20.45 | 2.64 | 16.59 | 0.825 | 45.27 | 154.1 |  |
| N-12 | 13.17 | 36.83 | 1.54 | 39.95 | 0.758 | 1.42 | 34.3 | -60 |
| N-13 | 15.57 | 33.67 | 1.84 | 35.52 | 0.774 | 2.35 | 60.1 | -60 |
| N-14 | 16.70 | 32.84 | 1.98 | 33.93 | 0.78 | 2.93 | 69.2 | -58 |
| N-15 | 17.70 | 32.21 | 1.94 | 30.81 | 0.783 | 3.33 | 66.7 | -60 |
| N-16 | 18.78 | 30.30 | 2.08 | 29.87 | 0.79 | 4.49 | 81.1 | -52 |
| N-17 | 20.00 | 29.50 | 2.12 | 27.80 | 0.799 | 5.67 | 88.2 | -47 |
| N-18 | 20.43 | 28.61 | 2.20 | 27.76 | 0.799 | 6.99 | 98.7 | -40 |
| N-19 | 23.52 | 27.10 | 2.32 | 23.95 | 0.806 | 10.04 | 105 | -44 |
| N-20 | 23.60 | 26.25 | 2.34 | 23.99 | - | - | - | -41 |
| N-21 | 25.26 | 25.74 | 2.50 | 23.07 | 0.81 | 11.76 | 125.5 | -34 |
| N-22 | 24.56 | 25.54 | 2.40 | 23.21 | 0.809 | 12.43 | 125.1 | -31 |
| N-23 | 24.83 | 25.25 | 2.42 | 23.02 | 0.81 | 13.33 | 124.8 | -36 |
| N-24 | 26.76 | 24.15 | 2.50 | 21.42 | 0.813 | 15.62 | 134.4 | -33 |
| N-25 | 29.88 | 23.72 | 2.88 | 20.71 | 0.814 | 17.94 | 139 | -30 |
| N-26 | 29.95 | 23.23 | 2.64 | 19.34 | 0.816 | 19.66 | 139.2 | -27 |
| N-27 | 32.97 | 21.03 | 2.70 | 17.46 | 0.823 | 33.87 | 151.8 | -35 |

Table S4 Data Sheet of Structural Parameters and Physicochemical Properties of FT Lubricant Base Stocks

| No. | C* | BI/% | B | BI*/% | Density /g.cm^-3^ | 40℃ viscosity /mm^2^.s^-1^ | Viscosity index | Condensation point,/℃ |
| --- | --- | --- | --- | --- | --- | --- | --- | --- |
| L-1 | 29.37 | 28.01 | 4.46 | 31.24 | 0.794 | 17.04 | 170.4 |  |
| L-2 | 28.6 | 27.85 | 4.46 | 32.45 | 0.792 | 6.61 | 154.54 | -45 |
| L-3 | 27.61 | 31.91 | 4.22 | 32.26 | 0.784 | 5.06 | 187.6 | -48 |
| L-4 | 22.74 | 23.4 | 2.16 |  | 0.798 | 7.44 | 110.3 | -57 |
| L-5 | 33.63 | 25.94 | 4.42 | 25.68 | 0.802 | 13.4 | 143.86 | -47 |
| L-6 | 29.55 | 28.01 | 4.5 | 31.25 | 0.7958 | 8.3 | 182.62 |  |
| L-7 | 32.18 | 30.31 | 4.7 | 28.97 | 0.788 | 7.79 | 122.05 | -39 |
| L-8 | 30.4 | 22.66 | 4.76 | 31.80 | 0.812 | 18.76 | 137 | -37 |
| L-9 | 35.17 | 28.92 | 4.76 | 25.97 | 0.822 | 31.41 | 104.1 | -46 |
| L-10 | 34.65 | 25.05 | 4.64 | 25.85 | 0.806 | 18.02 | 148.3 | -34 |
| L-11 | 30.05 | 32.07 | 4.02 | 27.34 | 0.782 | 8.23 | 204.61 | -52 |

**5．Validation data**

Table S5 Data structure table

|  | Pt | Pb | PN | PI | CP | BI | B | C* |
| --- | --- | --- | --- | --- | --- | --- | --- | --- |
| 1 | 6.82 | 7.02 | 47.78 | 53.88 | 101.67 | 21.79 | 2.06 | 29.80 |
| 2 | 7.23 | 6.94 | 49.75 | 56.10 | 105.84 | 26.04 | 1.92 | 29.30 |
| 3 | 5.72 | 5.20 | 37.28 | 43.76 | 81.04 | 22.15 | 1.82 | 28.30 |
| 4 | 7.24 | 7.96 | 49.77 | 68.73 | 118.50 | 26.88 | 2.48 | 32.75 |
| 5 | 6.58 | 8.09 | 53.50 | 55.68 | 109.18 | 26.46 | 2.46 | 33.19 |
| 6 | 9.57 | 10.72 | 35.69 | 72.47 | 108.16 | 26.25 | 2.24 | 22.61 |
| 7 | 9.15 | 10.06 | 32.85 | 76.64 | 109.48 | 23.31 | 2.20 | 23.93 |
| 8 | 8.37 | 9.87 | 38.66 | 55.63 | 94.30 | 26.25 | 2.36 | 22.54 |
| 9 | 9.46 | 11.26 | 29.71 | 69.32 | 99.02 | 29.37 | 2.38 | 20.93 |
| 10 | 8.76 | 8.76 | 42.33 | 56.11 | 98.44 | 23.72 | 2.00 | 22.47 |

**6.** **Formula Promotion**

Table S6 Comparative Performance Data

|  | Oil Name | Structure index | Calculated Viscosity/ mm^2^·s^-1^ | Viscosity at 40°C/ mm^2^·s^-1^ | Calculated Viscosity Index | Viscosity Index | Calculated condensation point/℃ | Condensation point/℃ |
| --- | --- | --- | --- | --- | --- | --- | --- | --- |
| F-Toil | CTL4  ^[1]^ | 108 | 5.6 | 3.94 | 121.7 | 128 | -16 | -33 |
|  | YU4 ^[1]^ | 100 | 5.2 | 4.12 | 115.3 | 119 | -20 | -21 |
|  | GTL4 ^[1]^ | 89 | 4.6 | 4.063 | 155 | 121 | -26 | -39 |
| PAO | Mpao4 ^[1]^ | 110 | 5.7 | 3.85 | 173.1 | 125 | -55 | -75 |
|  | PAO4-M ^[1]^ | 75 | 3.9 | 4.12 | 140.2 | 122 | -73 | -66 |
|  | Trimer 1-Octene^[2]^ | 48 | 2.5 | 2.05 | 101.6 | 111 | -87 | -75 |
|  | Tetramer 1-Octene^[2]^ | 78 | 4 | 3.56 | 143.1 | 123 | -72 | -57 |
|  | Trimer 1-Decene^[2]^ | 56 | 2.9 | 0.58 | 114.8 | 142 | -83 | -66 |
|  | Tetramer 1-Decene^[2]^ | 90 | 4.6 | 5.94 | 155.4 | 146 | -66 | -54 |
|  | Trimer 1-Dodecene^[2]^ | 64 | 3.3 | 4.67 | 126.2 | 150 | -79 | -55 |
|  | PAO-2^[3]^ | 45 | 2.3 | 1.9 | 96 | - | -88 | -70 |
|  | PAO-4^[3]^ | 65 | 3.3 | 4.06 | 127.5 | 124 | -78 | -68 |
| Mineral oil | oil S-9^[3]^ | - | - | 2.74 | - | 86 | - | -50 |
|  | MS-8 base^[3]^ stock^[3]^ | - | - | 2.74 | - | 90 | - | -55 |
|  | oil AU^[3]^ | - | - | 2.82 | - | 41 | - | -48 |
|  | oil GK^[3]^ | - | - | 3.4 | - | 94 | - | -60 |
|  | NB 3020^[3]^ | - | - | 2.24 | - | 99 | - | -54 |
|  | VHVI-4^[3]^ | - | - | 4.43 | - | 123 | - | -24 |

[1] Liu, J., Zhang, Z., Zhou, X., Hu, W., Pan, R. and Li, J., 2024. Structure–Performance Relationship of Coal-Based Lubricating Base Oils and Sensitivities to Typical Additives. Lubricants, 12(5), p.156.

[2] Xue, J.Y., Dong, S.Q., Mi, P.K., Wang, L.B., Wang, S.H., Zhang, Z., Zhang, Z.G. and Hu, J.S., 2021. Study of the structure-activity relationship of metallocene-catalysed poly-α-olefin (mPAO) base oil. Molecular Systems Design & Engineering, 6(9), pp.722-729.

[3] Porfiryev, Y., Shuvalov, S., Popov, P., Kolybelsky, D., Petrova, D., Ivanov, E., Tonkonogov, B. and Vinokurov, V., 2020. Effect of base oil nature on the operational properties of low-temperature greases. ACS omega, 5(21), pp.11946-11954.

[4] Gross, A.S., 2022. Simple Physics-Guided Compositional Model for Kinematic Viscosity at 100°C for Groups II and III Base Oils and Base Stocks. Industrial & Engineering Chemistry Research, 61(25), pp.9087-9098.

**7. Technical methods for gas phase analysis**

The ASTM-7169 standard is referenced, which is a test method that utilises high temperature gas chromatography to determine the boiling point distribution and carbon number distribution of lubricant base oils. A flame ionisation detector is used as a data acquisition system operating in slice mode. A data acquisition system operating in slice mode and chromatography software are used to accumulate the electronic signals. Retention time calibration mixtures were used to plot retention time versus boiling point. The solvent feed subtracts the resulting signal from the response factor standard chromatogram and the sample chromatogram. The standard and sample chromatograms are subtracted from each other. The final sample solution is injected and the amount of sample recovered is calculated using the response factor. The amount of sample recovered is calculated. After converting the retention time of the sample sections to temperature, the boiling point distribution can be calculated up to the amount of sample recovered. Boiling point distribution up to the amount recovered. Individual components of the sample are eluted from the gas chromatography in the order of the boiling point, and the sample is injected into the programmed warming inlet port in the autosampler, and warmed up according to the method procedure, so that the sample is gradually introduced into the GC column from the low to the high boiling point, and the column separates hydrocarbon compounds according to the order of the boiling point, which leads to the distribution of the sample's boiling point and carbon number range.


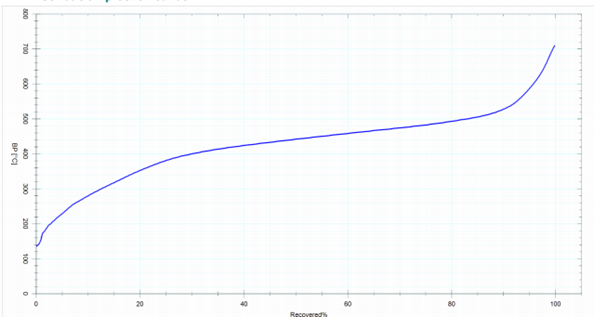


Figure S3 Distribution of distillation ranges obtained by high-temperature gas phase using L-1 as an example

Figure S4 Carbon number distribution obtained by high-temperature gas phase using L-1 as an example


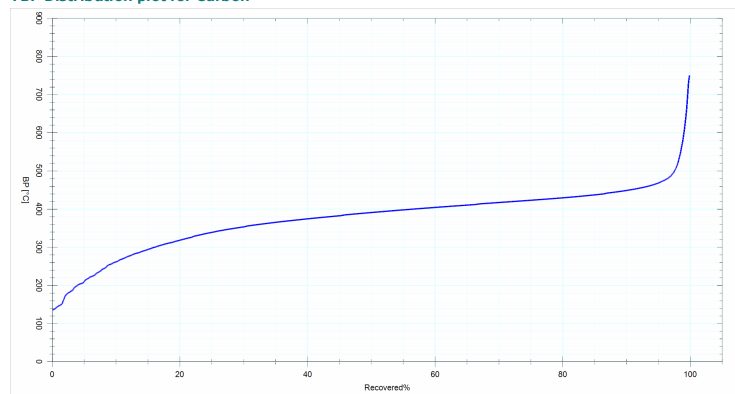


Figure S5 Distribution of distillation ranges obtained by high-temperature gas phase using L-2 as an example


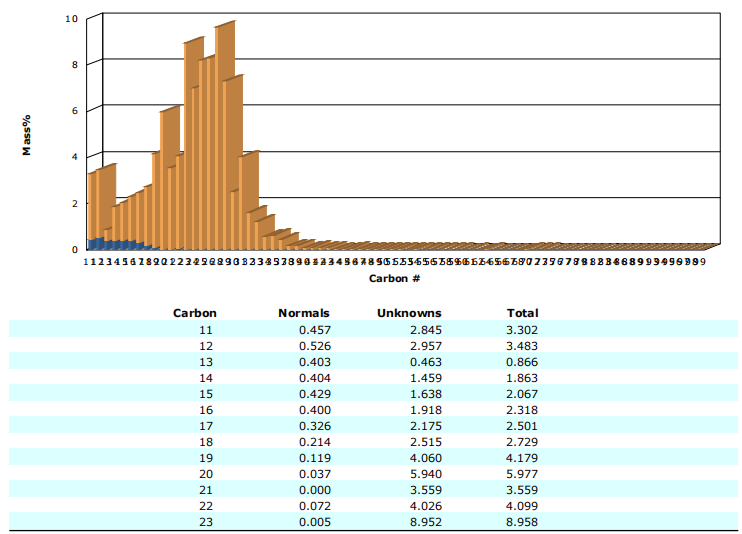

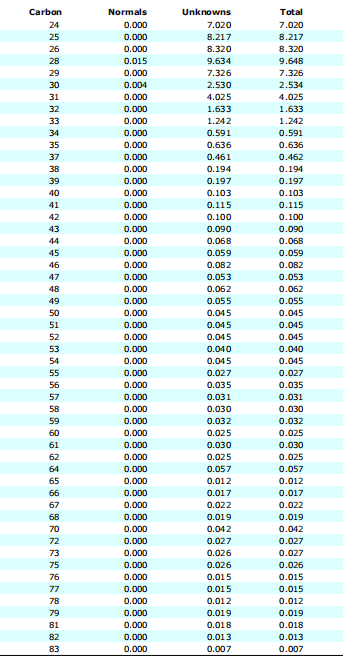

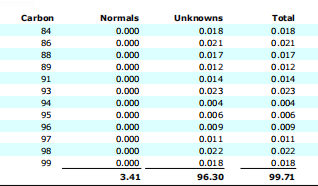


Figure S6 Carbon number distribution obtained by high-temperature gas phase using L-2 as an example


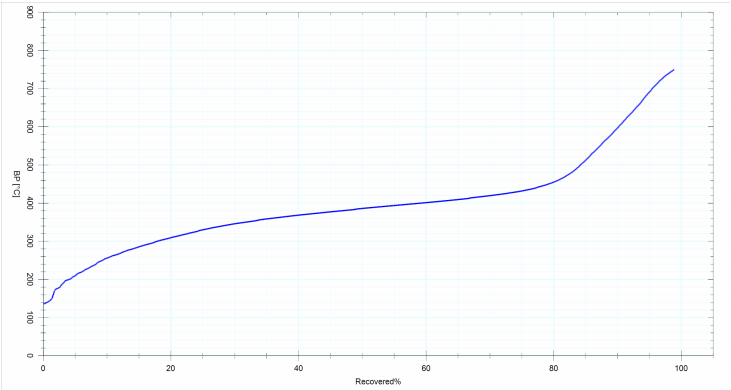


Figure S7 Distribution of distillation ranges obtained by high-temperature gas phase using L-3 as an example


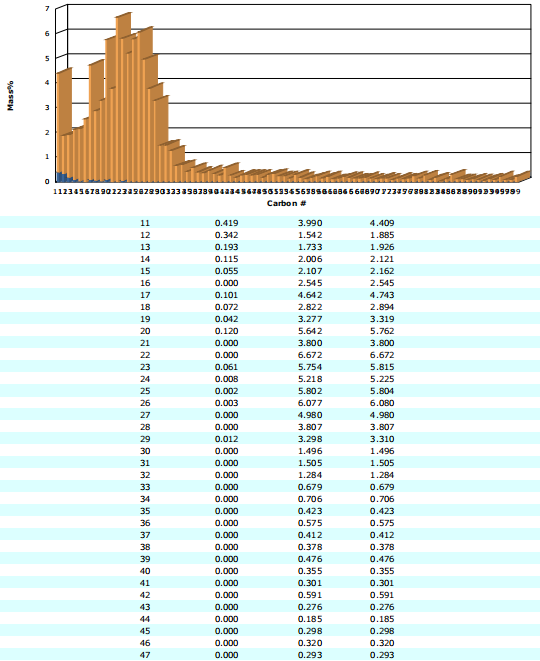

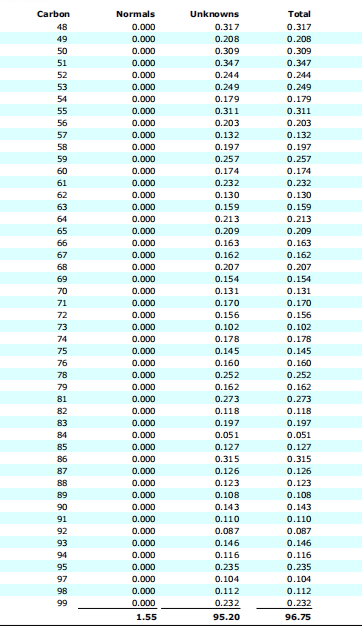


Figure S8 Carbon number distribution obtained by high-temperature gas phase using L-3 as an example
